# Residual left atrial v wave predicts clinical outcome of transcatheter edge-to-edge mitral valve repair

**DOI:** 10.1093/eschf/xvag086

**Published:** 2026-03-23

**Authors:** Michael Paulus, Jonas Rösch, Franziska Grewe, Moritz Haus, Valeska Bienert, Michael Wester, Christian Schach, Andreas Luchner, Christoph Birner, Bernhard Unsöld, Lars S Maier, Kurt Debl, Christine Meindl

**Affiliations:** Department of Internal Medicine II, University Hospital Regensburg, Franz-Josef-Strauß-Allee 11, Regensburg 93053, Germany; Department of Internal Medicine II, University Hospital Regensburg, Franz-Josef-Strauß-Allee 11, Regensburg 93053, Germany; Department of Internal Medicine II, University Hospital Regensburg, Franz-Josef-Strauß-Allee 11, Regensburg 93053, Germany; Department of Internal Medicine II, University Hospital Regensburg, Franz-Josef-Strauß-Allee 11, Regensburg 93053, Germany; Department of Internal Medicine II, University Hospital Regensburg, Franz-Josef-Strauß-Allee 11, Regensburg 93053, Germany; Department of Internal Medicine II, University Hospital Regensburg, Franz-Josef-Strauß-Allee 11, Regensburg 93053, Germany; Department of Internal Medicine II, University Hospital Regensburg, Franz-Josef-Strauß-Allee 11, Regensburg 93053, Germany; Department of Cardiology, Hospital Barmherzige Brüder Regensburg, Regensburg, Germany; Department of Internal Medicine I, Klinikum St.Marien, Amberg, Germany; Department of Internal Medicine II, University Hospital Regensburg, Franz-Josef-Strauß-Allee 11, Regensburg 93053, Germany; Medical Clinic I, Cardiology and Angiology, Justus-Liebig-University Giessen, Giessen, Germany; Department of Internal Medicine II, University Hospital Regensburg, Franz-Josef-Strauß-Allee 11, Regensburg 93053, Germany; Department of Internal Medicine II, University Hospital Regensburg, Franz-Josef-Strauß-Allee 11, Regensburg 93053, Germany; Department of Internal Medicine II, University Hospital Regensburg, Franz-Josef-Strauß-Allee 11, Regensburg 93053, Germany

**Keywords:** Mitral regurgitation, Edge-to-edge repair, Left atrial pressure, V wave

## Abstract

**Background and Aims:**

Intraprocedural assessment of residual mitral regurgitation (MR) is crucial for the success of transcatheter edge-to-edge mitral valve repair (M-TEER), yet challenging in the case of ambiguous echocardiographic findings. Monitoring left atrial (LA) pressure can complement the evaluation of residual MR after device placement. This study aimed to determine the prognostic impact of intraprocedural changes in LA pressure on the clinical outcome following M-TEER.

**Methods:**

We enrolled 299 patients undergoing M-TEER for primary or secondary MR in a prospective observational study. During the procedure, LA mean (LAmP) and LA v wave pressure (LAvP) were recorded before and after device implantation. The primary endpoint was death or hospitalization for heart failure during a 2-year follow-up.

**Results:**

Mean age of the study population was 76.6 ± 8.2 years. Secondary mitral regurgitation was identified in 62.9% of the patients. Reduction to MR grade I or II was achieved in 95.3% of cases. During M-TEER, LAvP decreased from 30.5 ± 15.0 to 23.2 ± 10.4 mmHg (*P* < .001) after device implantation, accompanied by a modest reduction of LAmP from 16.6 ± 6.3 to 15.3 ± 5.9 mmHg (*P* = .006). LAvP post M-TEER was a strong predictor of death or hospitalization for heart failure in both univariate and multivariate analysis, independent of echocardiographic MR severity (hazard ratio per 10 mmHg 1.37 [1.15–1.63], *P* < .001 and 1.29 [1.06–1.57], *P* = .012). Residual LAvP below 25 mmHg was strongly associated with a favourable outcome irrespective of residual echocardiographic MR grade, including patients with residual MR grade I and II.

**Conclusion:**

High residual LAvP predicts death or hospitalization for heart failure after M-TEER. LAvP after device implantation provides incremental prognostic information beyond echocardiographic MR grading and may therefore assist intraprocedural decision-making during M-TEER.

## Graphical Abstract

**Graphical Abstract:**
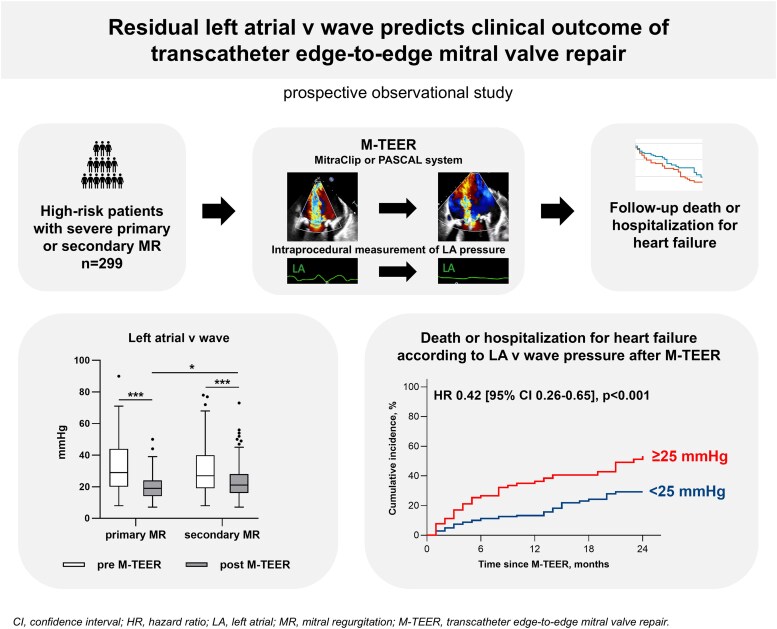

## Introduction

Achieving adequate reduction of mitral regurgitation (MR) is the primary objective of transcatheter edge-to-edge mitral valve repair (M-TEER) and determines the clinical outcome of the procedure.^1,2^ Transoesophageal echocardiography remains the primary modality for evaluation of immediate MR reduction during the intervention, guiding clinical decisions on device positioning and deployment. However, due to methodical limitations or suboptimal image quality, echocardiographic assessment may remain ambiguous in cases of more than mild residual MR, requiring integration of additional clinical parameters.^3,4^ Modern M-TEER systems allow for invasive measurement of left atrial (LA) pressure by transducing the side port of the guide catheter, enabling clinicians to evaluate LA haemodynamics before and after device implantation, as well as continuously throughout the procedure.^5^ An immediate decrease in LA pressure following device placement is indicative of successful MR reduction and can serve as a valuable complement to echocardiography, thereby enhancing procedural decision-making.^6,7^ Previous studies have demonstrated that both LA mean pressure (LAmP) at baseline and after M-TEER are correlated with the clinical outcome of the intervention.^8,9^ However, elevation of LAmP is not specific to MR, but the result of various conditions which lead to elevated left ventricular filling pressure.^10,11^ High v waves in the LA pressure waveform are regarded as a classical and more specific sign of significant MR^12^ and can be readily detected through pressure monitoring during M-TEER. Nonetheless, the prognostic significance of LA v wave pressure (LAvP) after device implantation remains uncertain. Based on these considerations, we aimed to systematically investigate the intraprocedural changes in LAvP and their relationship with long-term clinical outcomes following M-TEER.

## Methods

### Study design

From August 2017 to March 2024, patients were consecutively enrolled in a prospective observational study conducted at the University Heart Center Regensburg. The primary inclusion criterion for enrolment was the presence of symptomatic moderate-to-severe or severe MR treated with M-TEER. Patients with intraprocedural failure to implant a device (*n* = 4) or incomplete data on LA pressure (*n* = 12) were excluded from the study. The decision to proceed with M-TEER was made on an individual basis by an interdisciplinary Heart Team. All participants underwent a thorough clinical and echocardiographic evaluation prior to the procedure. MR grading was based on colour and continuous wave Doppler examination in accordance with current guidelines.^13,14^ Regurgitation grade was scored from I to IV (I: mild, II: mild-to-moderate, III: moderate-to-severe, IV: severe). Following the procedure, patients were observed for up to 24 months to assess the incidence of a composite endpoint consisting of death or hospitalization for heart failure. Informed consent was obtained from all participants. The study complied with the Declaration of Helsinki and was approved by the local ethics committee.

### Procedure

M-TEER procedures were conducted by experienced interventionalists utilizing either the MitraClip (Abbott Vascular, Menlo Park, USA) or PASCAL system (Edwards Lifesciences, Irvine, USA) under general anaesthesia with guidance from fluoroscopy and three-dimensional transoesophageal echocardiography. Following transseptal puncture and advancement of the guide sheath into the LA, pressure curves were recorded to measure LAmP and LAvP using the side port of the catheter. LAvP was calculated as the mean value across three cardiac cycles. In cases of atrial fibrillation, LAvP was measured during a single cardiac cycle immediately following two consecutive similar R-R intervals (index beat method). To evaluate LA pressure post-procedure, measurements were repeated at the conclusion of the procedure before withdrawing the guide sheath through the interatrial septum. Before each measurement, vasopressor support was titrated to achieve a systemic pressure target corresponding to the patient's preprocedural blood pressure.

### Statistical analysis

Continuous variables are reported as mean ± standard deviation when normally distributed, continuous variables with skewed distribution as median with interquartile range [first quartile-third quartile]. Categorical variables are presented in numbers and percentages. Differences in unpaired data were assessed using Student’s *t*-test for normally distributed data, Mann–Whitney *U* test for ordinal or skewed data, Pearson’s chi-squared test for nominal data, and Fisher’s exact test for dichotomous data. Differences in paired samples were tested using paired t-tests for normally distributed data, Wilcoxon signed-rank tests for skewed or ordinal data, and McNemar’s test for dichotomous data. To identify predictors of death or hospitalization for heart failure, univariate and multivariate Cox regression models were calculated. Due to substantial correlations among the haemodynamic parameters and moderate multicollinearity (variance inflation factors 3.4–4.3), these variables were not entered simultaneously into a single multivariable Cox model. Instead, five separate multivariable models were constructed, each including one haemodynamic parameter at a time, while adjusting for the same set of clinical covariates. Kaplan-Meier analysis was performed to compare the incidence of death or hospitalization for heart failure between groups. Receiver operating characteristic analysis (ROC) with calculation of the Youden index was used to identify the optimal cutoff value of haemodynamic parameters in predicting the clinical endpoint. Binary logistic regression analysis was performed to identify predictors of elevated postprocedural LA pressure. Owing to its skewed distribution, NTproBNP was included in the model as its natural logarithm. A two-sided *P*-value of <.05 was considered statistically significant. All statistical analyses were performed using SPSS Statistics 29 (IBM, Armonk, USA).

## Results

### Baseline characteristics and procedural outcome

A total of 299 patients were enrolled in the study. Baseline characteristics are detailed in *Table 1*. Most participants presented with MR grade IV, accounting for 76.9% of the study population. Secondary mitral regurgitation was identified in 62.9% of the patients. M-TEER was performed using one device in 57.5% of cases, while two devices were utilized in 39.8% of procedures (*Table 2*). At the time of discharge, effective reduction of mitral regurgitation was achieved in most patients, with 95.3% attaining MR grade I or II. The mean mitral valve pressure gradient observed at discharge was 3.6 ± 1.5 mmHg.

**Table 1:** Baseline characteristics of the study population

	All (*n* = 299)	LAvP post M-TEER		*P*-value
		<25 mmHg (*n* = 209)	≥25 mmHg (*n* = 90)	
Age, years	76.6 ± 8.2	76.5 ± 8.1	76.9 ± 8.3	.677
Female gender	110 (36.8)	74 (35.4)	36 (40.0)	.514
BMI, kg/m^2^	26.2 ± 5.0	26.1 ± 4.8	26.3 ± 5.5	.693
Coronary artery disease	183 (61.2)	81 (38.8)	35 (38.9)	1.000
Atrial fibrillation	202 (67.6)	134 (64.1)	68 (75.6)	.060
Diabetes mellitus	77 (25.8)	47 (22.5)	30 (33.3)	.061
CRT	21 (7.0)	13 (6.2)	8 (8.9)	.461
ICD	45 (15.1)	28 (13.4)	27 (18.9)	.223
GFR, ml/min	48.5 ± 20.6	49.8 ± 20.5	45.4 ± 20.5	.**045**
NTproBNP, pg/ml	2221 [1064–4864]	1974 [881–3772]	3589 [1571–7503]	.**018**
NYHA functional class				.140
I	5 (1.7)	4 (1.9)	1 (1.1)	
II	72 (24.1)	55 (26.3)	17 (18.9)	
III	199 (66.6)	135 (64.6)	64 (71.1)	
IV	23 (7.7)	15 (7.2)	8 (8.9)	
Six-minute walk distance, m	267 [174–340]	270 [197–346]	220 [146–339]	.119
Secondary MR	188 (62.9)	126 (59.8)	63 (70.0)	.117
MR grade				.**043**
III	69 (23.1)	55 (26.3)	14 (15.6)	
IV	230 (76.9)	154 (73.7)	76 (84.4)	
LVEF, %	47.4 ± 14.4	47.6 ± 14.7	47.0 ± 13.9	.757
LVEDD, mm	56.8 ± 9.4	56.5 ± 9.1	57.6 ± 10.1	.341
LA volume index, ml/m^2^	83.1 ± 38.6	82.8 ± 37.6	83.7 ± 40.8	.864
RV basal diameter, mm	39.2 ± 7.2	38.5 ± 7.1	40.9 ± 7.3	.**010**
TAPSE, mm	18.7 ± 4.2	18.9 ± 4.1	18.2 ± 4.3	.162
Tricuspid regurgitation grade				.**009**
Mild	87 (29.1)	68 (32.5)	10 (21.1)	
Moderate	104 (34.8)	75 (35.9)	29 (32.2)	
Severe	108 (36.1)	66 (31.6)	42 (46.7)	
Peak TRV, cm/s	326 ± 55	320 ± 54	339 ± 54	.**007**
E/e’	14.8 ± 5.4	14.0 ± 4.9	16.6 ± 6.1	.**001**

Variables are expressed as *n* (%), mean ± standard deviation, or median [interquartile range], as appropriate. Boldface *P*-values denote statistical significance (*P*<0.05).

BMI, body mass index; CRT, cardiac resynchronization therapy; GFR, glomerular filtration rate; ICD, implantable cardioverter-defibrillator; LA, left atrial; LAvP, left atrial v wave pressure; LV, left ventricular; LVEDD, left ventricular end diastolic diameter; LVEF, left ventricular ejection fraction; MR, mitral regurgitation; MV, mitral valve; NYHA, New York Heart Association; RV, right ventricle; TAPSE, tricuspid annular plane systolic excursion; TRV, tricuspid regurgitation velocity.

**Table 2:** Procedural characteristics of the study population

	All (*n* = 299)	LAvP post M-TEER		*P*-value
		<25 mmHg (*n* = 209)	≥25 mmHg (*n* = 90)	
No. of implanted devices				.**044**
1	172 (57.5)	126 (60.3)	46 (51.1)	
2	119 (39.8)	83 (39.7)	36 (40.0)	
3	8 (2.7)	0	8 (8.9)	
Device type				.134
MitraClip	206 (68.9)	138 (66.0)	68 (75.6)	
PASCAL	93 (31.1)	71 (34.0)	22 (24.4)	
MR grade at discharge				.**027**
I	175 (58.5)	129 (61.7)	46 (51.1)	
II	110 (36.8)	76 (36.4)	34 (37.8)	
III	12 (4.0)	4 (1.9)	8 (8.9)	
IV	2 (0.7)	0	2 (2.2)	
Mean MV pressure gradient at discharge, mmHg	3.6 ± 1.5	3.4 ± 1.5	3.8 ± 1.5	.**028**

Variables are expressed as *n* (%), mean ± standard deviation, or median [interquartile range], as appropriate. Boldface *P*-values denote statistical significance (*P*<0.05).

LAvP, left atrial v wave pressure; MR, mitral regurgitation; MV, mitral valve.

### Intraprocedural change in left atrial pressure

Following device implantation, immediate changes in LA pressures were observed. LAmP showed a modest decrease, shifting from 16.6 ± 6.3 mmHg before implantation to 15.3 ± 5.9 mmHg after the procedure (*P* = .006). This represented a median relative reduction of 5.0% [9.1–23.1%]. In contrast, the reduction in LAvP was more pronounced, declining from 30.5 ± 15.0 mmHg before device deployment to 23.2 ± 10.4 mmHg afterward (*P* < .001). This corresponded to a median relative reduction of 21.1% [5.7–42.3%].

**Figure 1:**
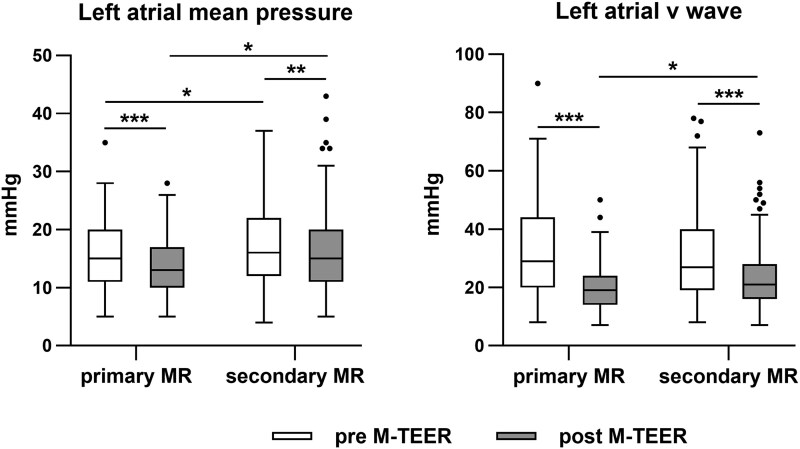
Intraprocedural LA pressure before and after M-TEER, stratified by MR aetiology. While the effects of M-TEER on LA mean pressure were only modest, LA v wave pressure substantially decreased after device implantation, with a median relative reduction of 21%. Reduction of LA pressure was observed in both patients with secondary and primary aetiology. Data are shown as Tukey-style box plots. *P < .05; ***P < .001. LA, left atrial; MR, mitral regurgitation; M-TEER, transcatheter edge-to-edge mitral valve repair

Both primary and secondary MR patients demonstrated reductions in LAmP and LAvP following device implantation (*Figure 1*). Notably, patients with secondary MR exhibited higher LAmP than those with primary MR, both before and after the procedure (before device: 17.2 ± 6.7 vs. 15.5 ± 5.5 mmHg, *P* = .025; after device: 16.1 ± 6.3 vs. 14.0 ± 4.9 mmHg, *P* = .003). While baseline LAvP measurements did not differ significantly between primary and secondary MR groups, secondary MR was associated with higher LAvP values following device implantation (23.2 ± 10.4 vs. 20.2 ± 8.5 mmHg, *P* = .011). Furthermore, the reduction in LAvP was less pronounced among secondary MR patients, with a relative reduction of 17.0% [4.9%–35.3%] versus a 31.8% [15.2%–52.2%] reduction in those with primary MR (*P* < .001).

### Impact of postprocedural left atrial pressure on clinical outcome

**Figure 2:**
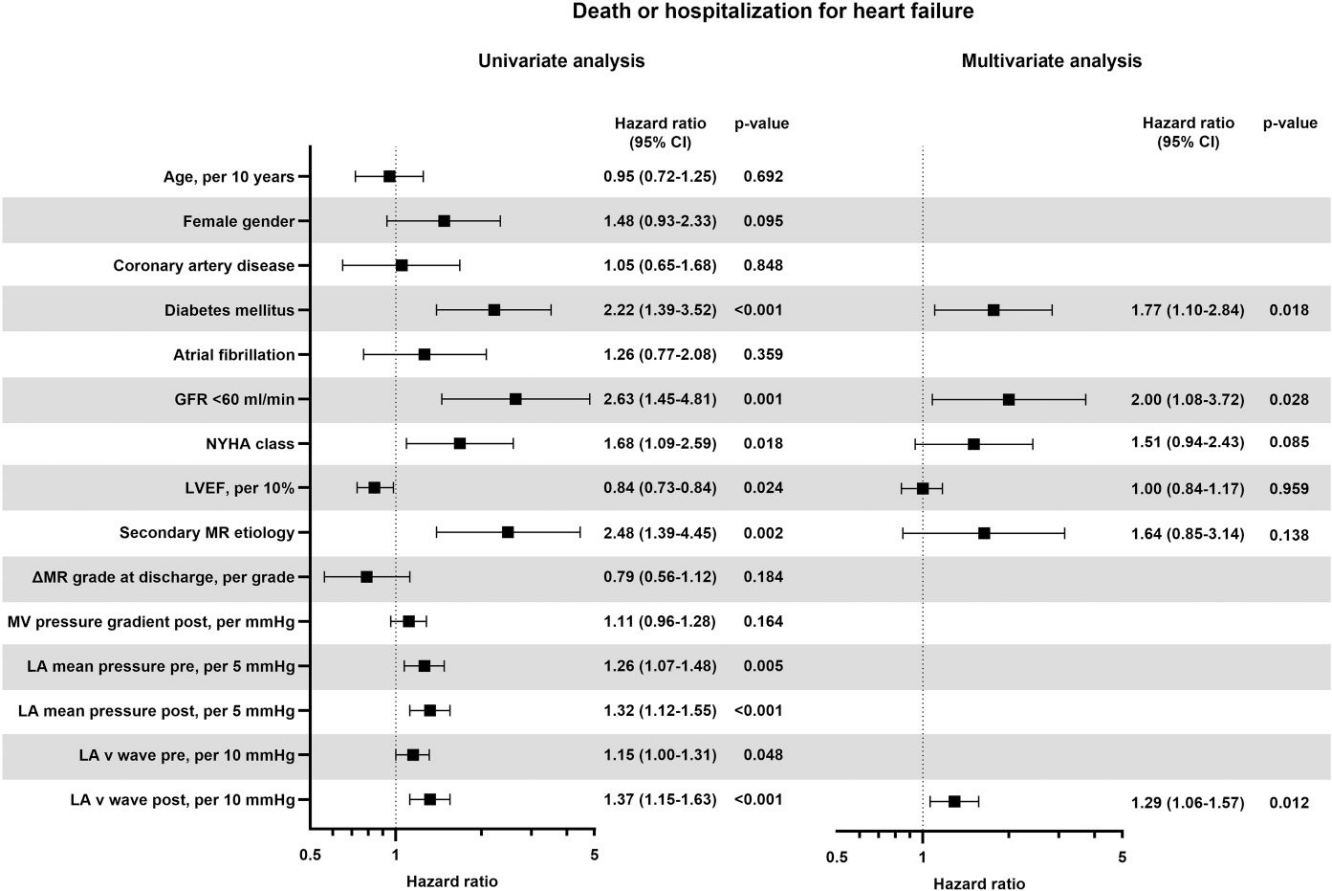
Predictors of death or hospitalization for heart failure after M-TEER. Higher LA pressure both before and after M-TEER was associated with worse prognosis in univariate analysis. LA v wave pressure after device implantation was the strongest haemodynamic predictor for postprocedural outcome, remaining independent of other clinical risk factors in multivariate analysis. CI, confidence interval; GFR, glomerular filtration rate; LA, left atrial; LVEF, left ventricular ejection fraction; MR, mitral regurgitation; MV, mitral valve; NYHA, New York Heart Association

To evaluate the association between LA pressure and clinical outcomes following M-TEER, we conducted an analysis focusing on predictors of a combined endpoint of death or hospitalization for heart failure. Median follow-up time was 12 months; the endpoint was reached in 24.7% of patients. The results of Cox proportional hazards regression are presented in *Figure 2*. Univariate analysis revealed that both elevated LAmP and increased LAvP—measured before and after device implantation—were significantly associated with a higher incidence of the combined endpoint. Among all haemodynamic parameters assessed, postprocedural LAvP emerged as the strongest predictor of death or hospitalization for heart failure. Specifically, each 10 mmHg increase in LAvP after M-TEER was linked to a 37% relative risk increase for the endpoint. Further, in a multivariate analysis adjusting for comorbidities and established clinical risk factors, the association remained robust. LAvP measured after device implantation continued to independently predict the combined endpoint, with a hazard ratio (HR) of 1.29 per 10 mmHg (95% confidence interval [CI] 1.06–1.57, *P* = .012).

Additional multivariable models revealed that postprocedural LAmP independently predicted the combined endpoint (HR per 5 mmHg 1.21 [95% CI 1.02–1.45], *P* = .032; *Supplementary Table S1*). In contrast, neither baseline LAvP nor LAmP served as independent predictors of mortality or hospitalization for heart failure. Notably, change in LAvP following M-TEER (ΔLAvP) also demonstrated a strong predictive value for the composite endpoint in multivariable analysis (HR per 10 mmHg 1.29 [95% CI 1.01–1.69], *P* = .041). Overall, these results underscore that the reduction in LA pressure after device implantation provides robust prognostic information regarding procedural outcomes, independent of baseline LA haemodynamics, MR aetiology, and heart failure characteristics.

**Figure 3:**
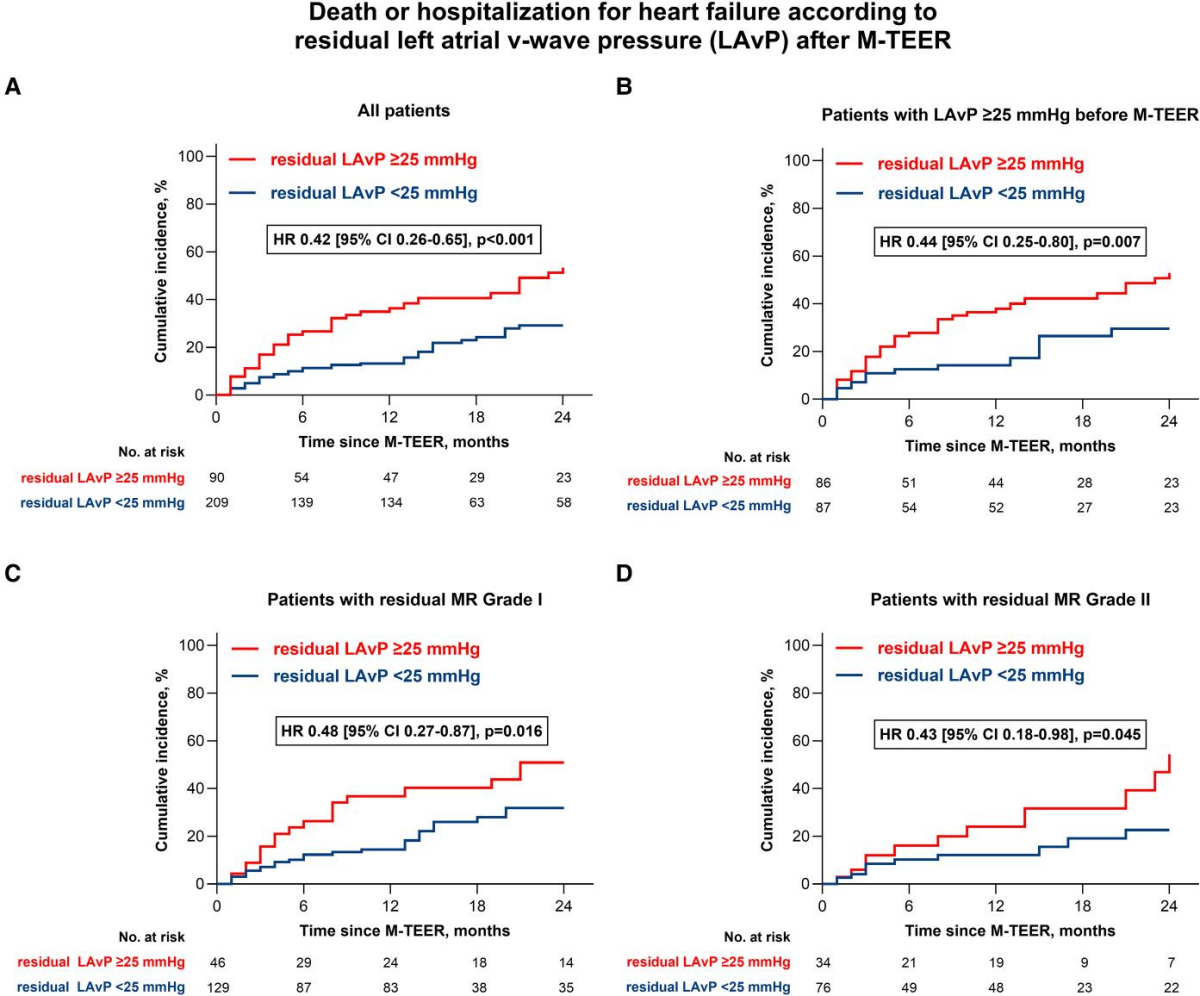
Death or hospitalization for heart failure after M-TEER according to postprocedural LA v wave pressure. Residual v wave below 25 mmHg was associated with superior long-term outcome. The prognostic effect was similarly observed in subgroups stratified by baseline v wave and postprocedural residual MR. CI, confidence interval; HR, hazard ratio; LA, left atrial; MR, mitral regurgitation; M-TEER, transcatheter edge-to-edge mitral valve repair

Based on ROC analysis (area under the curve 0.672, *P* < .001), the study population was stratified using a post-M-TEER LAvP cut-off value of 25 mmHg. Patients with post-implantation LAvP values below this threshold demonstrated a significantly reduced incidence of death or hospitalization for heart failure during follow-up (HR 0.42 [95% CI 0.26–0.65], *P* < .001; *Figure 3A*). Sensitivity analysis confirmed the predictive value of postprocedural LAvP across multiple subgroups. The threshold remained prognostically relevant in both secondary and primary MR (*Supplementary Figure S1*), as well as when excluding individuals without elevated LAvP prior to device implantation (*Figure 3B*). Importantly, maintaining a postprocedural LAvP below 25 mmHg was strongly associated with favourable outcomes, regardless of whether residual MR grade I or II was present (*Figs 3C* and *D*). Moreover, patients with residual MR grade II but LAvP below 25 mmHg experienced better outcomes compared to those with only mild residual MR but LAvP above the threshold (HR 0.36 [95% CI 0.17–0.76], *P* = .007). These results underscore that intraprocedural assessment of LA haemodynamics offered additional prognostic insight in both primary and secondary MR, complementing echocardiographic evaluation of MR reduction. An exploratory analysis of individual endpoints indicated that the observed difference in the composite outcome was attributable to similar reductions in both mortality (HR 0.41 [95% CI 0.20–0.85], *P* = .005) and hospitalization rates (HR 0.41 [95% CI 0.23–0.76], *P* = .002; *Supplementary Figure S2*).

Patients who had a residual LAvP of 25 mmHg or higher after M-TEER tended to require a greater number of devices, had more residual MR, and exhibited higher mitral valve pressure gradients at discharge. These features suggest lower procedural success and more complex mitral valve anatomy in this group (*Table 1*). Moreover, at baseline, these patients presented with more severe MR and tricuspid regurgitation, higher NTproBNP levels, and elevated systolic pulmonary artery pressures. For further sensitivity analysis, the association between LAvP ≥25 mmHg and the composite endpoint was examined across categories of left ventricular ejection fraction (LVEF) and postprocedural mitral valve gradient (*Supplementary Table S2*). The association remained directionally consistent across all LVEF subgroups, while not reaching statistical significance in patients with preserved LVEF, potentially due to the limited number of events. Similarly, the association between elevated LAvP and adverse outcomes was observed in both patients with low (≤4 mmHg) and elevated (>4 mmHg) residual mitral valve gradients. Importantly, no significant interaction was observed between LAvP and either LVEF category (*P* for interaction = .41) or mitral gradient strata (*P* for interaction = .52), suggesting that the prognostic relevance of elevated LAvP was consistent across heart failure phenotypes and independent of residual transmitral gradient.

### Characteristics of patients with high postprocedural left atrial pressure despite mild residual regurgitation

Following device implantation, 46 patients (15.3%) exhibited residual LAvP levels of 25 mmHg or greater in the setting of only mild residual MR. The characteristics of this subgroup, compared with patients displaying low residual LAvP, are detailed in *Supplementary Table S3*. Persistent elevation of LAvP after M-TEER despite optimal correction of MR was associated with secondary MR aetiology, poorer baseline NYHA functional class, elevated baseline NTproBNP levels, and increased left ventricular filling pressure indices before the procedure. Among these parameters, elevated baseline NTproBNP and E/e’ (odds ratio 1.71 [1.08–2.71], *P* = .022 and 1.09 [1.01–1.18], *P* = .026) were found to be independent predictors of LAvP ≥25 mmHg despite mild residual MR. Importantly, no significant differences were observed regarding postprocedural transmitral gradients, number of devices implanted, baseline LVEF or LA volumes.

## Discussion

This study systematically investigated LA haemodynamics during M-TEER and their prognostic implications. Its main findings are:

- While intraprocedural reduction of LAmP is only modest, device implantation is followed by an immediate and substantial reduction of LAvP which can be detected by continuous pressure monitoring.
- High residual LAvP strongly predicts death or hospitalization for heart failure after M-TEER in both primary and secondary MR.
- Patients with LAvP ≥25 mmHg after device implantation have a markedly increased risk for unfavourable clinical outcome, which persists even in cases where adequate MR reduction has been achieved.

### Prognostic significance of changes in left atrial pressure after M-TEER

Elevation of LA pressure accompanied by prominent or ‘giant’ v waves is a well-established haemodynamic hallmark of significant MR, reflecting atrial volume overload caused by the regurgitant flow.^15^ Multiple studies have demonstrated that successful M-TEER leads to an immediate reduction in LA pressure.^7,9,16,17^ Increasing evidence suggests that this haemodynamic response serves as a predictor for the long-term clinical outcome following M-TEER. Most previous research has focused on LAmP as the primary haemodynamic parameter of interest. Sammour et al. reported that in patients with primary MR, those whose LAmP post M-TEER fell within the higher two tertiles faced a significantly increased risk of death or hospitalization for heart failure. However, this association was not observed in secondary MR.^9^ Another multicentre study found that LAmP above 15 mmHg after M-TEER was strongly linked to worse prognosis.^18^ Comparable findings were reported by El Shaer et al., who identified a cut-off value of ≥22 mmHg for adverse outcomes.^19^ The current study supports these observations: High LAmP after device implantation was a strong predictor of unfavourable clinical outcome during follow-up. However, it is important to note that the immediate effects of M-TEER on LAmP may be modest. In the present analysis, the mean reduction in LAmP after device implantation was only about 1 mmHg. These subtle changes may be challenging to detect through continuous pressure monitoring, which might limit the utility of this haemodynamic parameter for real-time assessment of procedural success.

In contrast, immediate reduction of LAvP during M-TEER appears to be notably more pronounced. In this study, a median reduction of approximately 21% in LAvP was observed following device implantation, with the effect being especially significant in patients with primary MR. These acute changes in LAvP should be readily detectable through continuous pressure monitoring during the procedure, which may enhance its value for real-time procedural guidance and decision-making regarding device placement. Despite these observations, prior data on the prognostic significance of residual LAvP after M-TEER are scarce. A retrospective study involving 50 patients evaluated the association between intraprocedural reduction of LAvP and functional improvement post M-TEER. The findings indicated that each 5 mmHg decrease in LAvP after M-TEER was associated with a 49% higher likelihood of improvement in the six-minute walk distance.^16^ The present study demonstrates that residual LAvP following device implantation also serves as a robust predictor of long-term primary outcome, specifically death or hospitalization for heart failure. By applying a cut-off value of 25 mmHg, patients at high risk for adverse outcomes could be identified in both primary and secondary MR groups. Notably, achieving residual LAvP below 25 mmHg was also beneficial in the subgroup of patients with elevated baseline v wave. In the present study, each 10-mmHg increment in residual LAvP after device implantation was associated with a 29% increase in the likelihood of death or hospitalization for heart failure during follow-up. However, residual LAvP likely integrates several determinants of postprocedural risk, including residual regurgitant volume, LA compliance, left ventricular filling pressures, and transmitral gradients. Thus, LAvP should be interpreted primarily as a prognostic and integrative haemodynamic marker rather than a validated therapeutic endpoint.

### Haemodynamic mismatch after M-TEER—persistent high left atrial pressure despite successful reduction of mitral regurgitation

In the present study, 15.3% of patients exhibited a residual LAvP of 25 mmHg or higher after M-TEER, even though echocardiography revealed only mild MR. This subgroup experienced a significantly worse prognosis compared to patients whose LAvP decreased below 25 mmHg with similarly mild residual MR. Therefore, for these individuals, successful MR reduction did not correspond with a sufficient decrease in LA pressure. This distinctive haemodynamic profile has previously been described by Shibahashi et al., who introduced the term ‘haemodynamic mismatch.’ In their analysis of 1477 patients undergoing M-TEER, 302 individuals were found to have persistently elevated LAmP above 15 mmHg despite only mild residual MR. Consistent with our findings, their results showed that, although echocardiography suggested a favourable procedural result, persistent elevation of LA pressure was associated with a higher risk of death or hospitalization over five years.^18^ Notably, patients with elevated LA pressure despite mild residual MR had worse outcomes than those with moderate residual MR but normalized LA pressure, which was also observed in the results of our study. In summary, these findings strongly indicate that LA pressure after device implantation provides further prognostic information that extends beyond the echocardiographic assessment of MR reduction.

Several factors may contribute to the observed discrepancy between MR reduction and persistent elevation of LA pressure. First, high LAvP after device implantation could reflect an underestimation of residual MR by echocardiography in certain cases. However, while distinguishing between moderate and severe residual MR using echocardiography can be challenging, mild residual MR should be more reliably identified by the absence of broad or eccentric colour Doppler jets and flow convergence.^3^ More plausibly, sustained elevation of LAvP may be attributed to underlying left ventricular and LA dysfunction, which are not directly addressed by MR correction alone. Consistent with this consideration, our study found that elevated left ventricular filling pressure indices prior to M-TEER independently predicted high residual LAvP despite only mild residual MR. This finding is supported by a recent study involving 22 patients with primary MR which assessed haemodynamic changes after M-TEER using LA and left ventricular pressure-volume loops.^20^ It found that patients with LAvP of 20 mmHg or higher after M-TEER had increased chamber stiffness and impaired left ventricular relaxation—key features of heart failure with preserved ejection fraction (HFpEF). Additionally, atrial fibrillation and high body mass index were both associated with persistent LA pressure elevation following M-TEER,^18^ and these are strong risk factors for HFpEF. As such, this haemodynamic pattern may help identify patients who could benefit from optimal HFpEF therapy after M-TEER, particularly with the use of SGLT2 inhibitors. Consistent with the findings of Shibahashi et al.,^18^ our study demonstrated that patients exhibiting elevated LAvP following device implantation also presented with a higher postprocedural transmitral pressure gradient. Similar to MR, mitral stenosis is characterized by increased LA pressure accompanied by prominent v-waves, which result from a reduction in atrial compliance.^21^ Therefore, persistently elevated LAvP after device implantation, even when MR has been optimally reduced, may signal an excessive reduction in the mitral valve area. In such cases, the development of mitral stenosis can negate the intended haemodynamic benefits of MR elimination. Given these complexities, consideration of changes in LA pressure after device implantation may be used to complement the echocardiographic assessment of residual mitral valve area. This approach might be especially valuable in circumstances where anatomical planimetry is challenging due to image shadowing or when Doppler-derived transmitral gradients are significantly affected by fluctuations in heart rate.^22^

### Integration of left atrial pressure monitoring in intraprocedural decision-making

Transoesophageal echocardiography remains the primary modality for intraprocedural evaluation of residual MR following device implantation. The reduction in MR grade as assessed by echocardiography has been shown to predict both mortality and morbidity after M-TEER.^1,2^ However, intraprocedural echocardiography is not without limitations, particularly when interpreting findings suggestive of more than mild residual MR. Commonly used methods for native valvular regurgitation assessment, such as proximal isovelocity surface area, are not validated for MR assessment after M-TEER. Alternative approaches, such as the vena contracta area, can be technically demanding and require high image quality.^3^ Moreover, acoustic shadowing produced by the implantation catheter before device release may obscure residual regurgitation jets.

As a result, echocardiographic evaluation may remain inconclusive when residual MR is greater than mild following device placement. In such cases, assessment of changes in LA pressure can provide a valuable and complementary perspective for evaluating MR reduction and guiding intraprocedural decisions regarding device placement. Modern M-TEER systems facilitate continuous monitoring of LA pressure, which allows for real-time assessment throughout the procedure.^5^ Previous research suggests that continuous LA pressure monitoring can enhance echocardiographic outcomes of M-TEER. In a study of 86 patients, those who received continuous LA pressure monitoring had significantly greater MR reduction at discharge compared to those with measurements only before and after clip implantation, demonstrating the effectiveness of this approach.^23^

Several studies consistently demonstrated that residual mild MR after M-TEER is associated with better clinical outcomes and less likelihood of MR recurrence as compared to moderate residual MR.^24–27^ Therefore, if significant MR persists following device implantation, favourable haemodynamic parameters such as a low LAvP should not be interpreted as sufficient justification to accept a clearly suboptimal result. Instead, optimization of the echocardiographic outcome should be pursued, either by adjusting device position or by implanting an additional device. However, complex anatomical features may prevent further MR reduction by M-TEER. In such cases, the clinician must decide whether to accept the achieved result or to abort the intervention. In this study, it was shown that favourable clinical outcomes were still observed in patients with moderate residual MR after M-TEER, provided that LAvP dropped below 25 mmHg post-treatment. Thus, in situations where a reduction to mild residual MR cannot be achieved after device implantation, normalization of LA pressure may serve as an additional indicator that the regurgitant volume has been sufficiently reduced to provide clinical benefit for the patient. However, in cases of unsatisfactory results, transcatheter mitral valve replacement is a viable alternative for patients at high surgical risk. Notably, recent studies have demonstrated that percutaneous transseptal mitral valve replacement effectively treats MR with low complication rates, particularly in patients unsuitable for M-TEER.^28,29^

The intraprocedural endpoint of M-TEER is inherently defined by a trade-off: adding or repositioning of devices may further decrease residual MR, yet can also narrow the effective mitral orifice, heightening the risk for iatrogenic mitral stenosis. Doppler-derived mean transmitral gradient measurement via intraprocedural echocardiography is widely employed to assess the impact of device implantation on mitral valve area, given its expediency and general applicability.^30^ Nevertheless, the degree to which elevated transmitral gradients following M-TEER are tolerable without compromising clinical outcome remains subject to considerable debate. Earlier guidelines established a threshold of 5 mmHg to prevent functional mitral stenosis;^31^ however, recent studies have reported inconsistent findings regarding the relationship between transmitral gradient and clinical outcomes across different MR aetiologies.^25,27,32^ Additionally, the assessment of mitral valve area using transmitral gradient during procedures may be affected by altered loading conditions and heart rate, often underestimating haemodynamics when the patient is awake.^33^

Based on our study observations, evaluating changes in LAvP during M-TEER may serve as an additional metric when weighing further MR reduction against increasing mitral valve gradient. When echocardiographic evaluation reveals mild-to-moderate residual MR after implantation of the first device, analysing the magnitude and trajectory of LAvP following placement of a second device may be particularly insightful. A concurrent decrease in both residual MR and LAvP after the second device may suggest further haemodynamic benefit for the patient. Conversely, if the impact on residual MR is limited and no further lowering of LAvP occurs, the advantage of a second device may not compensate for the reduction in mitral valve area. Likewise, an increase of LAvP after device implantation should raise suspicion for significant stenosis. Persistently elevated LAvP despite only mild residual MR should prompt a systematic reassessment, focusing on ruling out underestimation of residual MR via echocardiography and excessive narrowing of the mitral valve area leading to functional mitral stenosis. Prospective studies are warranted to determine whether targeting combined echocardiographic and haemodynamic endpoints during M-TEER can improve clinical outcomes beyond imaging-guided approaches alone.

### Study limitations

This study may have limitations. Given the observational design, causality cannot be inferred, and it remains unknown whether actively targeting specific LAvP thresholds during M-TEER improves clinical outcomes. As the investigation was conducted at a single academic tertiary care centre, the results may not be fully generalizable to other institutions with different patient populations. Nonetheless, the characteristics and comorbidities of the patients in this cohort are comparable to those documented in multicentre registries for both secondary and primary mitral regurgitation,^34,35^ suggesting that the study population reflects real-world individuals treated with M-TEER in modern clinical practice.

LA pressure measurements in this study were obtained during M-TEER performed under general anaesthesia and positive pressure ventilation, both of which are known to substantially alter cardiac loading conditions. Therefore, there are important limitations that need to be considered when interpreting our findings. General anaesthesia reduces systemic vascular resistance, venous return, and ventricular filling pressures, and has been shown to underestimate the severity of MR compared with awake conditions.^36,37^ In addition, positive pressure ventilation increases intrathoracic pressure, thereby reducing venous return and LA preload, which may further lower measured LA pressures and alter v wave amplitude independently of residual regurgitant volume.^38^ Moreover, experimental and clinical data indicate that general anaesthesia and mechanical ventilation impair LA mechanics and compliance, potentially affecting the morphology of the LA pressure waveform. Consequently, absolute LA pressure values obtained during the procedure may not fully reflect haemodynamics in the conscious state. However, the present study primarily focused on relative intraprocedural changes and postprocedural thresholds assessed under standardized procedural conditions, which preserves the internal validity of the observed associations with clinical outcome. Assessing LA pressure in a conscious state would have required separate right heart catheterization, which was beyond the scope of this research.

## Conclusions

High LAvP after device implantation is an independent predictor of mortality or hospitalization for heart failure following M-TEER in patients with both primary and secondary MR. Residual LAvP below 25 mmHg is correlated with improved clinical outcomes, both in the setting of mild or moderate residual MR. Monitoring changes in LA pressure after device implantation may offer insights into the prognostic effect of the achieved MR reduction, thereby assisting intraprocedural decision-making in scenarios with complex anatomy or ambiguous echocardiographic findings.

## Supplementary Material

xvag086_Supplementary_Data

## Declarations

### Data Availability

The data underlying this article will be shared on reasonable request to the corresponding author.

## Pre-registered Clinical Trial Number

None supplied.

## References

[1] Higuchi S, Orban M, Stolz L, Karam N, Praz F, Kalbacher D, et al Impact of residual mitral regurgitation on survival after transcatheter edge-to-edge repair for secondary mitral regurgitation. JACC Cardiovasc Interv 2021;14:1243–53. 10.1016/j.jcin.2021.03.050 33992551

[2] Kar S, Mack MJ, Lindenfeld J, Abraham WT, Asch FM, Weissman NJ, et al Relationship between residual mitral regurgitation and clinical and quality-of-life outcomes after transcatheter and medical treatments in heart failure: COAPT trial. Circulation 2021;144:426–37. 10.1161/CIRCULATIONAHA.120.053061 34039025

[3] Zoghbi WA, Asch FM, Bruce C, Gillam LD, Grayburn PA, Hahn RT, et al Guidelines for the evaluation of valvular regurgitation after percutaneous valve repair or replacement: a report from the American society of echocardiography developed in collaboration with the society for cardiovascular angiography and interventions, Japanese society of echocardiography, and society for cardiovascular magnetic resonance. J Am Soc Echocardiogr 2019;32:431–75. 10.1016/j.echo.2019.01.003 30797660

[4] Boekstegers P, Hausleiter J, Schmitz T, Bufe A, Comberg T, Seyfarth M, et al Intraprocedural residual mitral regurgitation and survival after transcatheter edge-to-edge repair: prospective German multicenter registry (MITRA-PRO). JACC Cardiovasc Interv 2023;16:574–85. 10.1016/j.jcin.2022.12.015 36922044

[5] Tang GHL, Ong LY, Kaple R, Ramlawi B, Dutta T, Zaid S, et al Continuous invasive haemodynamic monitoring using steerable guide catheter to optimize mitraclip transcatheter mitral valve repair: a multicenter, proof-of-concept study. J Interv Cardiol 2018;31:907–15. 10.1111/joic.12557 30168203

[6] Alkhouli M, Eleid MF, Nishimura RA, Rihal CS. The role of invasive hemodynamics in guiding contemporary transcatheter valvular interventions. JACC Cardiovasc Interv 2021;14:2531–44. 10.1016/j.jcin.2021.08.072 34887047

[7] Schmidt T, Schlüter M, Thielsen T, Alessandrini H, Schewel D, Kreidel F, et al Acute hemodynamic changes after mitraclip implantation comparing patients with degenerative and functional mitral regurgitation. Struct Heart 2017;1:188–94. 10.1080/24748706.2017.1358470

[8] Shaer E, Mahayni A, Simard A, Eleid T, Guerrero M, Rihal M, et al Baseline left atrial pressure predicts mortality following transcatheter edge-to-edge mitral valve repair. JACC Cardiovasc Interv 2021;14:2306–8. 10.1016/j.jcin.2021.07.057 34674870

[9] Sammour YM, Bou Chaaya RG, Hatab T, Zaid S, Aoun J, Makram OM, et al Impact of left atrial pressure on outcomes after mitral transcatheter edge-to-edge repair. Circ Cardiovasc Interv 2024;17:e014055. 10.1161/CIRCINTERVENTIONS.124.014055 38836574

[10] Park JS, Cho I, Kim D, Kim M-H, Park J-W, Yu HT, et al Differentiating left atrial pressure responses in paroxysmal and persistent atrial fibrillation: implications for diagnosing heart failure with preserved ejection fraction and managing atrial fibrillation. J Am Heart Assoc 2024;13:e035246. 10.1161/JAHA.124.035246 39189473 PMC11646497

[11] Guazzi M, Ghio S, Adir Y. Pulmonary hypertension in HFpEF and HFrEF. JACC Review Topic of the Week. J Am Coll Cardiol 2020;76:1102–11. 10.1016/j.jacc.2020.06.069 32854845

[12] Hayashi H, Abe Y, Morita Y, Nakane E, Haruna Y, Haruna T, et al The accuracy of a large V wave in the pulmonary capillary wedge pressure waveform for diagnosing current mitral regurgitation. Cardiology 2018;141:46–51. 10.1159/000493007 30317228

[13] Zoghbi WA, Adams D, Bonow RO, Enriquez-Sarano M, Foster E, Grayburn PA, et al Recommendations for noninvasive evaluation of native valvular regurgitation: a report from the American society of echocardiography developed in collaboration with the society for cardiovascular magnetic resonance. J Am Soc Echocardiogr 2017;30:303–71. 10.1016/j.echo.2017.01.007 28314623

[14] Praz F, Borger MA, Lanz J, Marin-Cuartas M, Abreu A, Adamo M, et al 2025 ESC/EACTS guidelines for the management of valvular heart disease. Eur Heart J 2025;46:4635–4736. 10.1093/eurheartj/ehaf194 40878295

[15] Pizzarello RA, Turnier J, Padmanabhan VT, Goldman MA, Tortolani AJ. Left atrial size, pressure, and V wave height in patients with isolated, severe, pure mitral regurgitation. Cathet Cardiovasc Diagn 1984;10:445–54. 10.1002/ccd.1810100505 6518508

[16] Maor E, Raphael CE, Panaich SS, Reeder GS, Nishimura RA, Nkomo VT, et al Acute changes in left atrial pressure after MitraClip are associated with improvement in 6-Minute walk distance. Circ Cardiovasc Interv 2017;10:e004856. 10.1161/CIRCINTERVENTIONS.116.004856 28314742

[17] Pierre K, Adedinsewo DA, Al-Hijji M, Miranda WR, Alkhouli M, Eleid MF, et al 30-day patient reported outcomes can be predicted by change in left atrial pressure and not change in transmitral gradient following MitraClip. Catheter Cardiovasc Interv 2021;97:1244–9. 10.1002/ccd.29477 33502087

[18] Shibahashi E, Yamaguchi J, Kawamoto T, Yoshikawa M, Kogure T, Inagaki Y, et al Mismatch between residual mitral regurgitation and left atrial pressure predicts prognosis after transcatheter edge-to-edge repair. JACC Cardiovasc Interv 2024;17:2126–37. 10.1016/j.jcin.2024.07.046 39322363

[19] Shaer E, Thaden A, Eleid J, Simard M, Guerrero T, Rihal M, et al Haemodynamic success is an independent predictor of mid-term survival after transcatheter edge-to-edge mitral valve repair. Circ Cardiovasc Interv 2022;15:e011542. 10.1161/CIRCINTERVENTIONS.121.011542 35176873

[20] Nagueh SF, Pournazari P, Wessly P, Hatab T, Bhimaraj A, Faza NN, et al Understanding the effects of mitral transcatheter edge-to-edge repair on left ventricular function using pressure-volume loops. JACC Adv 2025;4:101627. 10.1016/j.jacadv.2025.101627 39983617 PMC11889344

[21] Ha JW, Chung N, Jang Y, Kang WC, Kang SM, Rim SJ, et al Is the left atrial v. Wave the determinant of peak pulmonary artery pressure in patients with pure mitral stenosis? Am J Cardiol 2000;85:986–91. 10.1016/s0002-9149(99)00915-7 10760340

[22] Kassar M, Praz F, Hunziker L, Pilgrim T, Windecker S, Seiler C, et al Anatomical and technical predictors of three-dimensional mitral valve area reduction after transcatheter edge-to-edge repair. J Am Soc Echocardiogr 2022;35:96–104. 10.1016/j.echo.2021.08.021 34506920

[23] Horstkotte J, Kloeser C, Beucher H, Schwarzlaender E, von Bardeleben RS, Boekstegers P. Intraprocedural assessment of mitral regurgitation during the mitraclip procedure: impact of continuous left atrial pressure monitoring. Catheter Cardiovasc Interv 2016;88:1134–43. 10.1002/ccd.26504 27038227

[24] Makkar RR, Chikwe J, Chakravarty T, Chen Q, O’Gara PT, Gillinov M, et al Transcatheter mitral valve repair for degenerative mitral regurgitation. JAMA 2023;329:1778–88. 10.1001/jama.2023.7089 37219553 PMC10208157

[25] Tsunamoto H, Yamamoto M, Kagase A, Tokuda T, Sugiura A, Shimura T, et al Using transmitral pressure gradients and residual mitral regurgitation to optimize outcome after transcatheter edge-to-edge repair. J Am Coll Cardiol 2025;86:1684–700. 10.1016/j.jacc.2025.07.041 41193089

[26] Marcoff L, Koulogiannis K, Aldaia L, Mediratta A, Chadderdon SM, Makar MM, et al Echocardiographic outcomes with transcatheter edge-to-edge repair for degenerative mitral regurgitation in prohibitive surgical risk patients. JACC Cardiovasc Imaging 2024;17:471–85. 10.1016/j.jcmg.2023.09.015 38099912

[27] Singh GD, Price MJ, Shuvy M, Rogers JH, Grasso C, Bedogni F, et al Combined impact of residual mitral regurgitation and gradient after mitral valve transcatheter edge-to-edge repair. JACC Cardiovasc Interv 2024;17:2530–40. 10.1016/j.jcin.2024.08.004 39453373

[28] Groshenry N, Suc G, Mesnier J, Delhomme C, Cailliau A, Brochet E, et al Long-term clinical and hemodynamic outcomes of transcatheter mitral valve replacement. JACC Cardiovasc Interv 2026;19:239–51. 10.1016/j.jcin.2025.09.052 41605563

[29] Guerrero ME, Daniels DV, Makkar RR, Thourani VH, Asch FM, Pham M, et al Percutaneous transcatheter valve replacement in individuals with mitral regurgitation unsuitable for surgery or transcatheter edge-to-edge repair: a prospective, multicountry, single-arm trial. Lancet 2025;406:2541–50. 10.1016/S0140-6736(25)02073-2 41167201

[30] Biaggi P, Felix C, Gruner C, Herzog BA, Hohlfeld S, Gaemperli O, et al Assessment of mitral valve area during percutaneous mitral valve repair using the MitraClip system: comparison of different echocardiographic methods. Circ Cardiovasc Imaging 2013;6:1032–40. 10.1161/CIRCIMAGING.113.000620 24134955

[31] Stone GW, Adams DH, Abraham WT, Kappetein AP, Généreux P, Vranckx P, et al Clinical trial design principles and endpoint definitions for transcatheter mitral valve repair and replacement: part 2: endpoint definitions: a consensus document from the mitral valve academic research consortium. J Am Coll Cardiol 2015;66:308–21. 10.1016/j.jacc.2015.05.049 26184623

[32] Yoon S-H, Makar M, Kar S, Chakravarty T, Oakley L, Sekhon N, et al Prognostic value of increased mitral valve gradient after transcatheter edge-to-edge repair for primary mitral regurgitation. JACC Cardiovasc Interv 2022;15:935–45. 10.1016/j.jcin.2022.01.281 35512917

[33] Boerlage-van Dijk K, van Riel ACMJ, de Bruin-Bon RHACM, Wiegerinck EMA, Koch KT, Vis MM, et al Mitral inflow patterns after MitraClip implantation at rest and during exercise. J Am Soc Echocardiogr 2014;27:24–31.e1. 10.1016/j.echo.2013.09.007 24161483

[34] Ludwig S, Koell B, Weimann J, Donal E, Patel D, Stolz L, et al Impact of intraprocedural mitral regurgitation and gradient following transcatheter edge-to-edge repair for primary mitral regurgitation. JACC Cardiovasc Interv 2024;17:1559–73. 10.1016/j.jcin.2024.05.018 38986655

[35] Stocker TJ, Stolz L, Karam N, Kalbacher D, Koell B, Trenkwalder T, et al Long-term outcomes after edge-to-edge repair of secondary mitral regurgitation: 5-year results from the EuroSMR registry. JACC Cardiovasc Interv 2024;17:2543–54. 10.1016/j.jcin.2024.08.016 39537275

[36] Alachkar MN, Kirschfink A, Grebe J, Schälte G, Almalla M, Frick M, et al General anesthesia leads to underestimation of regurgitation severity in patients with secondary mitral regurgitation undergoing transcatheter mitral valve repair. J Cardiothorac Vasc Anesth 2022;36:974–82. 10.1053/j.jvca.2021.10.024 34799263

[37] Grewal KS, Malkowski MJ, Piracha AR, Astbury JC, Kramer CM, Dianzumba S, et al Effect of general anesthesia on the severity of mitral regurgitation by transesophageal echocardiography. Am J Cardiol 2000;85:199–203. 10.1016/s0002-9149(99)00644-x 10955377

[38] Corp A, Thomas C, Adlam M. The cardiovascular effects of positive pressure ventilation. BJA Educ 2021;21:202–9. 10.1016/j.bjae.2021.01.002 34026273 PMC8134774

