# Supplementary material for: Residual left atrial v wave predicts clinical outcome of transcatheter edge-to-edge mitral valve repair
Source: ESC Heart Fail. 2026 Mar 23;13(2):xvag086. doi: 10.1093/eschf/xvag086 (PMC13037373; doi:10.1093/eschf/xvag086)
Supplement: xvag086_Supplementary_Data [file xvag086_supplementary_data.docx]

Residual left atrial v wave predicts clinical outcome of transcatheter edge-to-edge mitral valve repair

Michael Paulus, Jonas Rösch, Franziska Grewe, Moritz Haus, Valeska Bienert, Michael Wester, Christian Schach, Andreas Luchner, Christoph Birner, Bernhard Unsöld, Lars S. Maier, Kurt Debl, Christine Meindl

Supplementary Data

**Address for correspondence:**

Michael Paulus

Department of Internal Medicine II

University Hospital Regensburg

Franz-Josef-Strauß-Allee 11, 93053 Regensburg, Germany

E-mail: michael.paulus@ukr.de

# Supplementary Figures


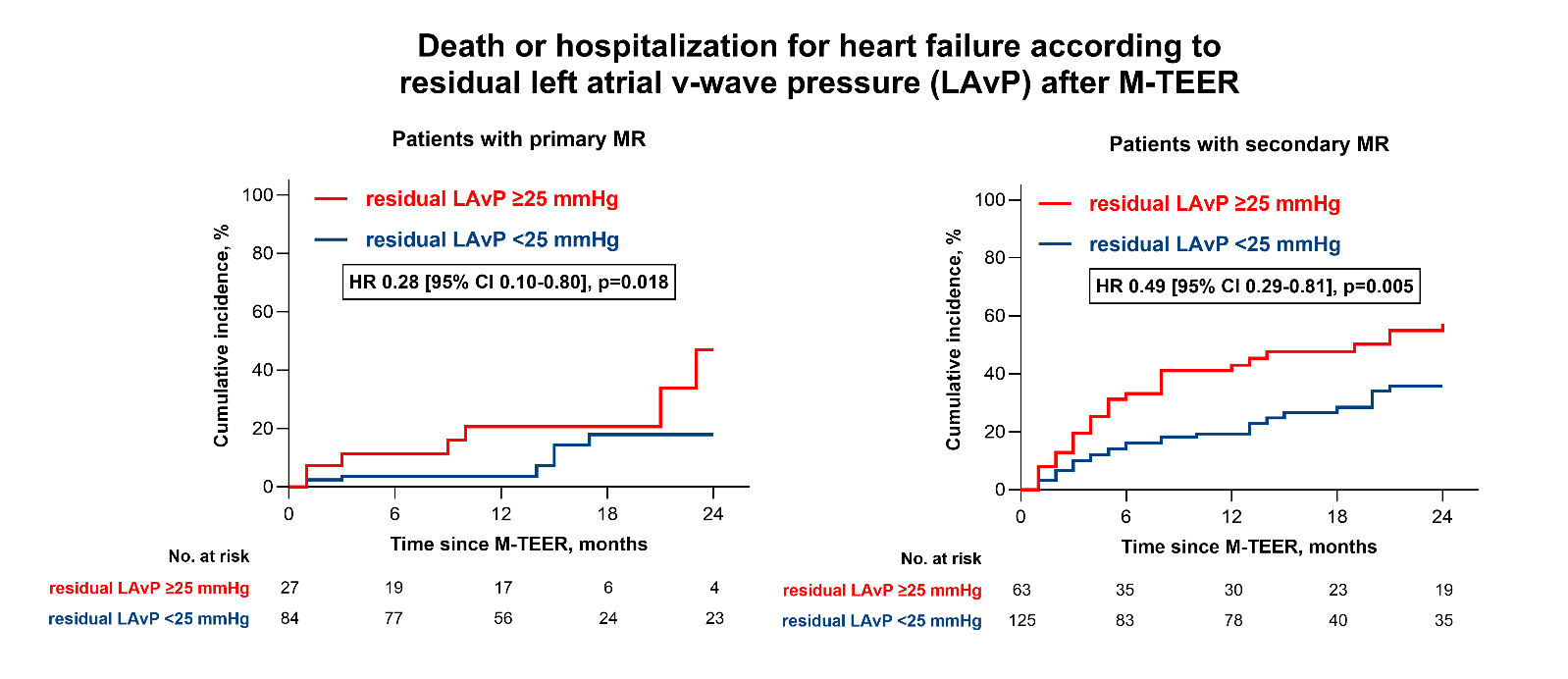


Supplementary Figure 1: Death or hospitalization for heart failure after edge-to-edge mitral valve repair in accordance to residual left atrial v wave pressure and mitral regurgitation etiology. *LA, left atrial; LAvP, left atrial v-wave pressure; MR, mitral regurgitation; M-TEER, transcatheter edge-to-edge mitral valve repair.*


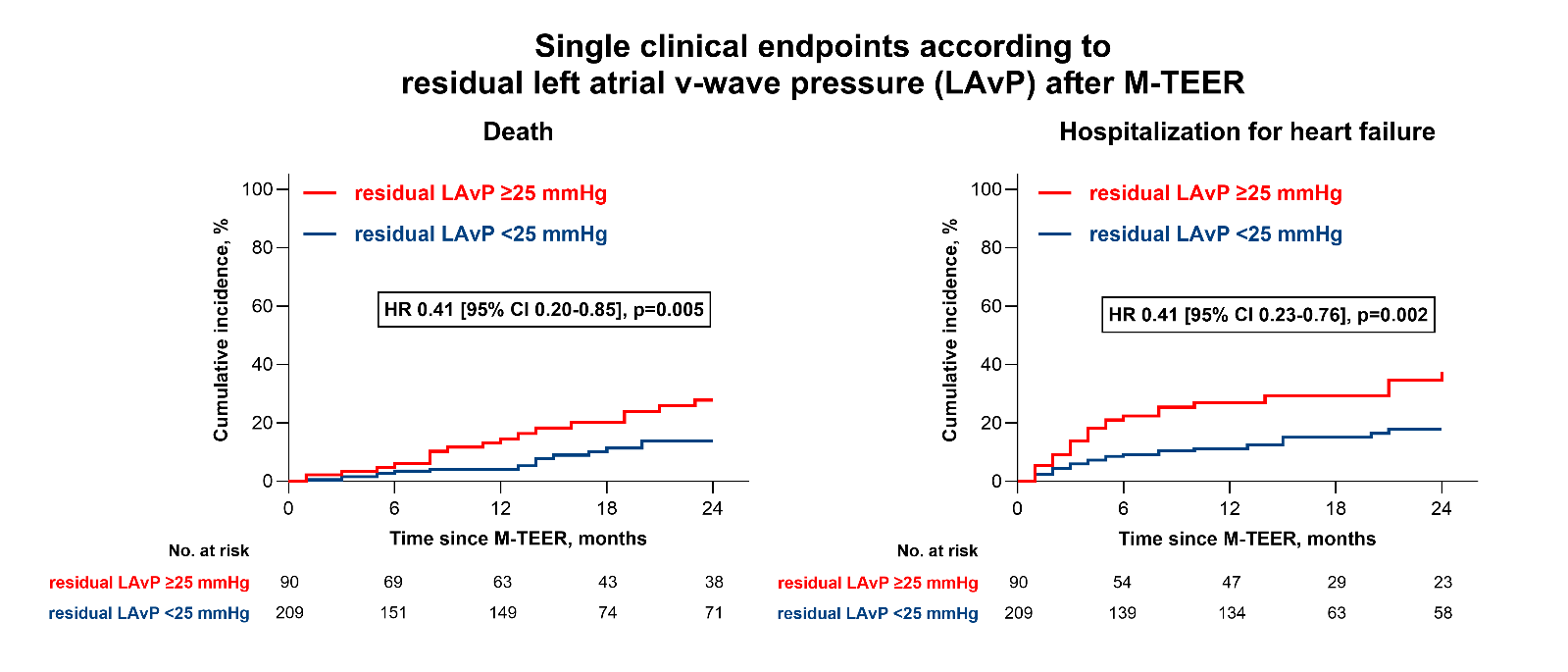


Supplementary Figure 2: Single clinical endpoints after edge-to-edge mitral valve repair in accordance to residual left atrial v wave pressure. *LA, left atrial; LAvP, left atrial v-wave pressure; M-TEER, transcatheter edge-to-edge mitral valve repair.*

# Supplementary Tables

Supplementary Table 1: Hemodynamic parameters in multivariable Cox regression for death or hospitalization for heart failure after edge-to-edge mitral valve repair.

| **Subgroup** | **Model 1**  **HR (95% CI)** | **Model 2**  **HR (95% CI)** | **Model 3**  **HR (95% CI)** | **Model 4**  **HR (95% CI)** | **Model 5**  **HR (95% CI)** |
| --- | --- | --- | --- | --- | --- |
| **Hemodynamic parameters** |  |  |  |  |  |
| LA mean pressure pre, per 5 mmHg | 1.15 (0.97-1.36) | - | - | - | - |
| LA mean pressure post, per 5 mmHg | - | 1.21 (1.02-1.45) | - | - | - |
| LA v wave pre, per 10 mmHg | - | - | 1.10 (0.83-1.16) | - | **1.28 (1.05-1.57)** |
| ∆LA v wave post, per 10 mmHg | - | - | - | **1.29 (1.06-1.57)** | **1.31 (1.01-1.69)** |
| **Clinical covariates** |  |  |  |  |  |
| Diabetes mellitus | **1.73 (1.07-2.79)** | **1.75 (1.09-2.80)** | **1.78 (1.10-2.86)** | **1.77 (1.10-2.84)** | **1.78 (1.11-2.87)** |
| GFR <60 ml/min | **1.98 (1.07-3.68)** | **2.01 (1.08-3.73)** | **1.95 (1.05-3.62)** | **2.00 (1.08-3.72)** | **2.01 (1.08-3.74)** |
| LVEF, per 10% | 0.99 (0.84-1.17) | 1.47 (0.92-2.35) | 0.98 (0.83-1.16) | 1.00 (0.84-1.17) | 1.00 (0.84-1.17) |
| Functional MR etiology | 1.80 (0.95-3.42) | 1.70 (0.89-3.25) |  | 1.64 (0.85-3.14) | 1.62 (0.83-3.14) |

Variables are expressed as n (%), mean±standard deviation, or median [interquartile range], as appropriate. *CI, confidence interval; GFR, glomerular filtration rate; HR, hazard ratio; LA, left atrial; LVEF, left ventricular ejection fraction; MR, mitral regurgitation.*

Supplementary Table 2: Risk for death or hospitalization for heart failure after edge-to-edge mitral valve repair in accordance to residual left atrial v wave pressure across subgroups.

| **Subgroup** | **events, n** | **HR [95% CI] for residual LAvP <25 mmHg** | **p-value** | **p-value for interaction** |
| --- | --- | --- | --- | --- |
| **Stratified by LVEF at baseline** |  |  |  | 0.754 |
| ≥50% (n=163) | 28 | 0.56 [0.26-1.18] | 0.134 |  |
| 41-49% (n=47) | 16 | 0.36 [0.14-0.97] | 0.044 |  |
| ≤40% (n=89) | 30 | 0.35 [0.17-0.71] | 0.004 |  |
| **Stratified by postprocedural mean mitral valve gradient** |  |  |  |  |
| >4 mmHg (n=63) | 20 | 0.35 [0.14-0.86] | 0.022 | 0.674 |
| ≤4 mmHg (n=236) | 54 | 0.45 [0.27-0.77] | 0.005 |  |

Variables are expressed as n (%), mean±standard deviation, or median [interquartile range], as appropriate. *CI, confidence interval; HR, hazard ratio; LA, left atrial; LAvP, left atrial v-wave pressure; LVEF, left ventricular ejection fraction.*

Supplementary Table 3: Clinical and procedural characteristics of patients with mild residual mitral regurgitation after edge-to-edge repair (n=175) in accordance to post-procedural left atrial v wave pressure.

|  | **LAvP after device implantation** | |  |
| --- | --- | --- | --- |
|  | **<25 mmHg**  **(n=129)** | **≥25 mmHg**  **(n=46)** | **p-value** |
| **Age, years** | 76.0±7.9 | 76.8±6.5 | 0.553 |
| **Female gender** | 45 (34.9) | 21 (45.7) | 0.218 |
| **BMI, kg/m²** | 26.1±4.7 | 26.7±6.7 | 0.525 |
| **Coronary artery disease** | 83 (64.3) | 31 (67.4) | 0.857 |
| **Atrial fibrillation** | 86 (66.7) | 31 (67.4) | 1.000 |
| **Diabetes mellitus** | 30 (23.3) | 17 (37.0) | 0.083 |
| **GFR, ml/min** | 46.9±20.0 | 45.7±20.0 | 0.731 |
| **NTproBNP, pg/ml** | 2039 [845-3974] | 3854 [1592-6499] | **0.002** |
| **NYHA functional class** |  |  | **0.014** |
| **I** | 3 (2.3) | 0 |  |
| **II** | 30 (23.3) | 3 (6.5) |  |
| **III** | 87 (67.4) | 39 (84.8) |  |
| **IV** | 9 (7.0) | 4 (8.7) |  |
| **Six-minute walk distance, m** | 259.8±108.6 | 220.1±123.1 | 0.075 |
| **Secondary MR** | 79 (61.2) | 37 (80.4) | **0.019** |
| **MR grade** |  |  | 0.143 |
| **III** | 43 (33.3) | 10 (21.7) |  |
| **IV** | 86 (66.7) | 36 (78.3) |  |
| **LVEF, %** | 48.0±13.9 | 46.6±13.7 | 0.553 |
| **LVEDD, mm** | 55.6±8.6 | 56.4±8.7 | 0.635 |
| **LA volume index, ml/m²** | 79.8±34.3 | 71.8±25.8 | 0.168 |
| **RV basal diameter, mm** | 38.3±7.4 | 40.5±7.0 | 0.084 |
| **TAPSE, mm** | 18.6±4.0 | 17.5±4.5 | 0.130 |
| **Tricuspid regurgitation grade** |  |  | 0.104 |
| **Mild** | 50 (38.8) | 12 (26.1) |  |
| **Moderate** | 37 (28.7) | 14 (30.4) |  |
| **Severe** | 42 (32.6) | 20 (43.5) |  |
| **Peak TRV, cm/s** | 316.6±55.0 | 335.9±55.5 | 0.050 |
| **E/e’** | 14.1±5.2 | 17.3±5.6 | **0.003** |
| **No. of implanted devices** |  |  |  |
| **1** | 82 (63.6) | 24 (52.2) | 0.124 |
| **2** | 47 (36.4) | 20 (43.5) |  |
| **3** | 0 | 2 (4.3) |  |
| **MR grade at discharge** |  |  | **-** |
| **I** | 129 (100.0) | 46 (100.0) |  |
| **Mean MV pressure gradient at discharge >4 mmHg** | 23 (17.8) | 10 (21.7) | 0.661 |

Variables are expressed as n (%), mean±standard deviation, or median [interquartile range], as appropriate. *BMI, body mass index; GFR, glomerular filtration rate; LA, left atrial; LAvP, left atrial v wave pressure; LV, left ventricular; LVEDD, left ventricular end diastolic diameter; LVEF, left ventricular ejection fraction; MR, mitral regurgitation; MV, mitral valve; NYHA, New York Heart Association; RV, right ventricle; TAPSE, tricuspid annular plane systolic excursion; TRV, tricuspid regurgitation velocity.*
